# Increased rate of cholecystectomies performed with doubtful or no indications after laparoscopy introduction: a single center experience

**DOI:** 10.1186/1471-2482-13-17

**Published:** 2013-05-31

**Authors:** Elia Pulvirenti, Adriana Toro, Michel Gagner, Maurizio Mannino, Isidoro Di Carlo

**Affiliations:** 1 Department of Surgical Sciences, Organ Transplantation and Advanced Technologies, University of Catania, Cannizzaro Hospital, Catania, Italy; 2 Department of Surgery, Herbert Wertheim College of Medicine, Florida International University, Miami, FL, USA; 3 Department of Surgical Sciences, Organ Transplantation, and Advanced Technologies, University of Catania, Cannizzaro Hospital, Via Messina 829, 95126, Catania, Italy

**Keywords:** Laparoscopic cholecystectomy, Open cholecystectomy, Gallbladder stones, Surgical indications, Laparoscopy

## Abstract

**Background:**

During recent years laparoscopic cholecystectomy has dramatically increased, sometimes resulting in overtreatment. Aim of this work was to retrospectively analyze all laparoscopic cholecystectomies performed in a single center in order to find the percentage of patients whose surgical treatment may be explained with this general trend, and to speculate about the possible causes.

**Methods:**

831 patients who underwent a laparoscopic cholecystectomy from 1999 to 2008 were retrospectively analyzed.

**Results:**

At discharge, 43.08% of patients were operated on because of at least one previous episode of biliary colic before the one at admission; 14.08% of patients presented with acute lithiasic cholecystitis; 14.68% were operated on because of an increase in bilirubin level; 1.56% were operated on because of a previous episode of jaundice with normal bilirubin at admission; 0.72% had gallbladder adenomas, 0.72% had cholangitis, 0.36% had biliodigestive fistula and one patient (0.12%) had acalculous cholecystitis. By excluding all these patients, 21.18% were operated on without indications.

**Conclusions:**

The broadening of indications for laparoscopic cholecystectomy is undisputed and can be considered a consequence of new technologies that have been introduced, increased demand from patients, and the need for practice by inexperienced surgeons. If not prevented, this trend could continue indefinitely.

## Background

Gallstones are a very common disease affecting up to 15% of the general population in the US and Europe [1], with a higher prevalence in women [2]. About 50 to 70% of patients with gallstones are asymptomatic [1] and nearly 1 in 10 individuals with asymptomatic gallstones may be expected to develop symptoms or complications that require treatment within five years [2].

Since its first introduction in the late 1980s, laparoscopic cholecystectomy has been extensively adopted as the treatment of choice in patients with gallstones and other less frequent benign gallbladder diseases [3] due to its undeniable advantages, such as the reduction of postoperative pain, faster recovery, and improved cosmesis [4]. Nevertheless, the undeniable advantages related to this procedure compared to the traditional open approach have resulted in a broadening of indications in performing such an operation, sometimes resulting in expensive and unnecessary overtreatment [4,5].

The aim of this work was to retrospectively analyze all patients who underwent a laparoscopic cholecystectomy in our department in order to find the percentage of patients whose surgical treatment may be explained with the general trend of increase in laparoscopy utilization, and to speculate about the possible causes.

## Methods

All patients who underwent a laparoscopic cholecystectomy at the Department of Surgical Sciences, Organ Transplantation and Advanced Technologies of University of Catania - Cannizzaro Hospital from 1999 to 2008 were retrospectively analyzed after approval of hospital ethics committee. Data were collected from medical records and patients were analyzed depending on the symptoms at admission and diagnosis at discharge in order to find the percentage of patients admitted and subsequently operated on with no indication. Patients operated with open technique and those who experienced a conversion to laparotomy were not considered for the present study.

Patient preoperative assessment was performed according the standard of care in our practice by routine laboratory tests and ultrasound (US). Computed tomography (CT) scan was performed only in cases of unclear diagnosis.

Patients who were diagnosed for gallstones at FAST and subsequently operated on at the resolution of the main cause of admission were also included in the present study.

Diagnosis of acute cholecystitis was based on the presence of right upper quadrant pain with or without fever and with evidence of raised WBC count, ultrasonographic evidence of gallstones, pericholecystic fluid collection, gall bladder wall thickness and thick wall contracted gall bladder.

According to the evidences in literature [6-11], conditions that were considered correct indications for surgery were the following: acute cholecystitis (including complicated form such as gallbladder perforation and gangrene), acute biliary pancreatitis, gallbladder adenomas, cholangitis with evidence of gallstones, biliodigestive fistula and Mirizzi syndrome, lithiasis of the biliary ducts with or without jaundice, recurrent episodes of biliary colic or recurrent jaundice and the rare Bouveret syndrome. Conversely, medical records of patients who were admitted only because of the general diagnosis of abdominal pain with gallstones, or patients without symptoms in which diagnosis was performed incidentally during FAST exam for trauma were further analyzed in order to find the percentage of patients operated on without indication.

The following parameters were considered: sex, age, presence of comorbidities (diabetes mellitus, hepatic cirrhosis, and immunosuppression), pre-and postoperative stay, length of hospitalization, history of previous biliary colic or jaundice, symptoms referred at the admission, diagnostic imaging performed, white blood cell count and bilirubin levels at the admission.

Patients with gallstones and vague abdominal pain with atypical localization or characteristics, who underwent an operation for gallbladder stones without previous episodes of pain, jaundice, increase in WBC count or bilirubin were considered as operated on without indication. These patients were subsequently contacted by telephone and an oral questionnaire was administered in order to assess the percentage of those who experienced early (within 30 days) or delayed recurrence of symptoms, as well as those who had no improvement in symptoms despite the operation.

### Local ethics committee who gave approval for the study

Cannizzaro Hospital Ethics Commettee.

## Results

From 1999 to 2008, 987 patients underwent a cholecystectomy. Among these, 105 patients (10.64%) underwent a primary open operation and 51 patients (5.17%) experienced a conversion to laparotomy after a laparoscopic attempt, so these 156 patients were excluded from the study. Among the remaining 831 patients, 495 (59.57%) were female. The main characteristics of these patients are shown in Table 1. Twenty-nine (3.49%) patients were diabetic and 20 (2.41%) were cirrhotic. The median age (25-75th percentile) was 57 years (43-68).

**Table 1:** Characteristics of the whole population of study

Number of patients	831
Females (%)	495 (59.57%)
Males (%)	336 (40.43%)
Median age in years (25-75th perc)	57 (43-68)
Cirrhotics (%)	20 (2.41%)
Diabetics (%)	29 (3.49%)
Immunodepressed (%)	1 (0.12%)
Median time to surgery in days (25-75th perc)	2 (1-5)
Median postoperative stay in days (25-75th perc)	2 (1-3)
Median overall stay in days (25-75th perc)	4 (2-8)
Previous episodes of biliary colic (%)	374 (45%)
Previous episodes of jaundice (%)	37 (4.45%)
Median WBC at admission in 10^9^ cells/L (25-75th perc)	8.1 (6.3-10.4)
Median bilirubin at admission in mg/100 mL (25-75th perc)	0.93 (0.69- 1.49)
Preoperative computed tomography (%)	43 (5.17%)

**Figure 1:**
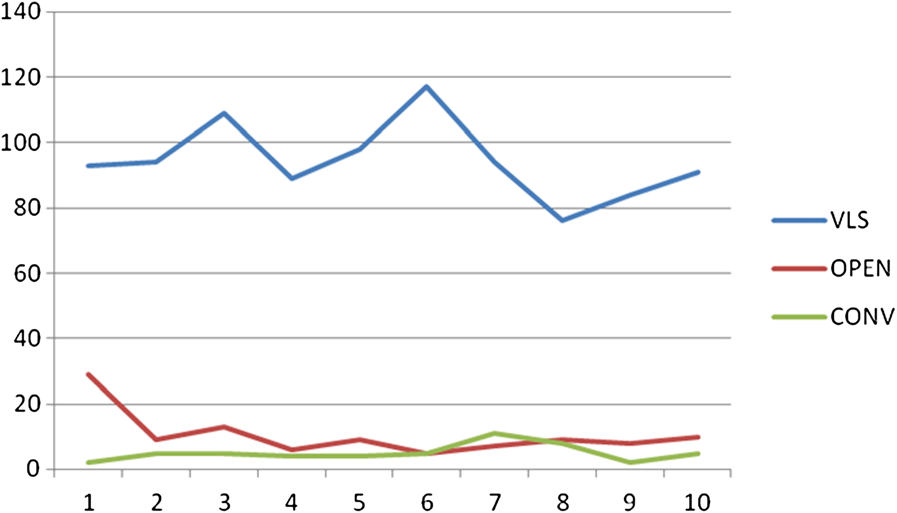
**The operative trend during the nine-year period of study, with regard to the approaches performed.** The study started when the laparoscopic cholecystectomy had already become the standard of care at our Department, replacing the open approach in most uncomplicated patients. A residual of the previous trend is evident at the beginning of the 1999, when the percentage of open cholecystectomy was higher. VLS: laparoscopic cholecistectomies; OPEN: open cholecystectomies; CONV: conversions.

The operative trend during the nine-year period with regard to the approaches performed is shown in Figure 1 and Table 2, which compare the number of laparoscopic cholecystectomies performed with open cholecystectomies and conversions to laparotomy. The study started when the laparoscopic cholecystectomy had already become the standard of care in our practice, replacing the open approach in most uncomplicated patients. At the beginning of 1999, the percentage of open cholecystectomy was higher, as a residual of the previous trend.

**Table 2:** Operative data during the nine-year period of study

**Year**	**Open**	**Conv**	**VLS**	**Tot**
**1999**	29	2	82	113
**2000**	9	5	83	97
**2001**	13	5	98	116
**2002**	6	4	78	88
**2003**	9	4	87	100
**2004**	5	5	102	112
**2005**	7	11	83	101
**2006**	9	8	65	82
**2007**	8	2	73	83
**2008**	10	5	80	95
Tot	105	51	831	987

Median time (25-75th percentile) from admission to operation was 2 (1-5) days, and median (25-75th percentile) postoperative stay was 2 (1-3) days with a median (25-75th percentile) overall hospital stay of 4 (2-8) days.

The main causes of admission followed by surgery were: abdominal pain, jaundice, acute pancreatitis and abdominal trauma.

**Figure 2:**
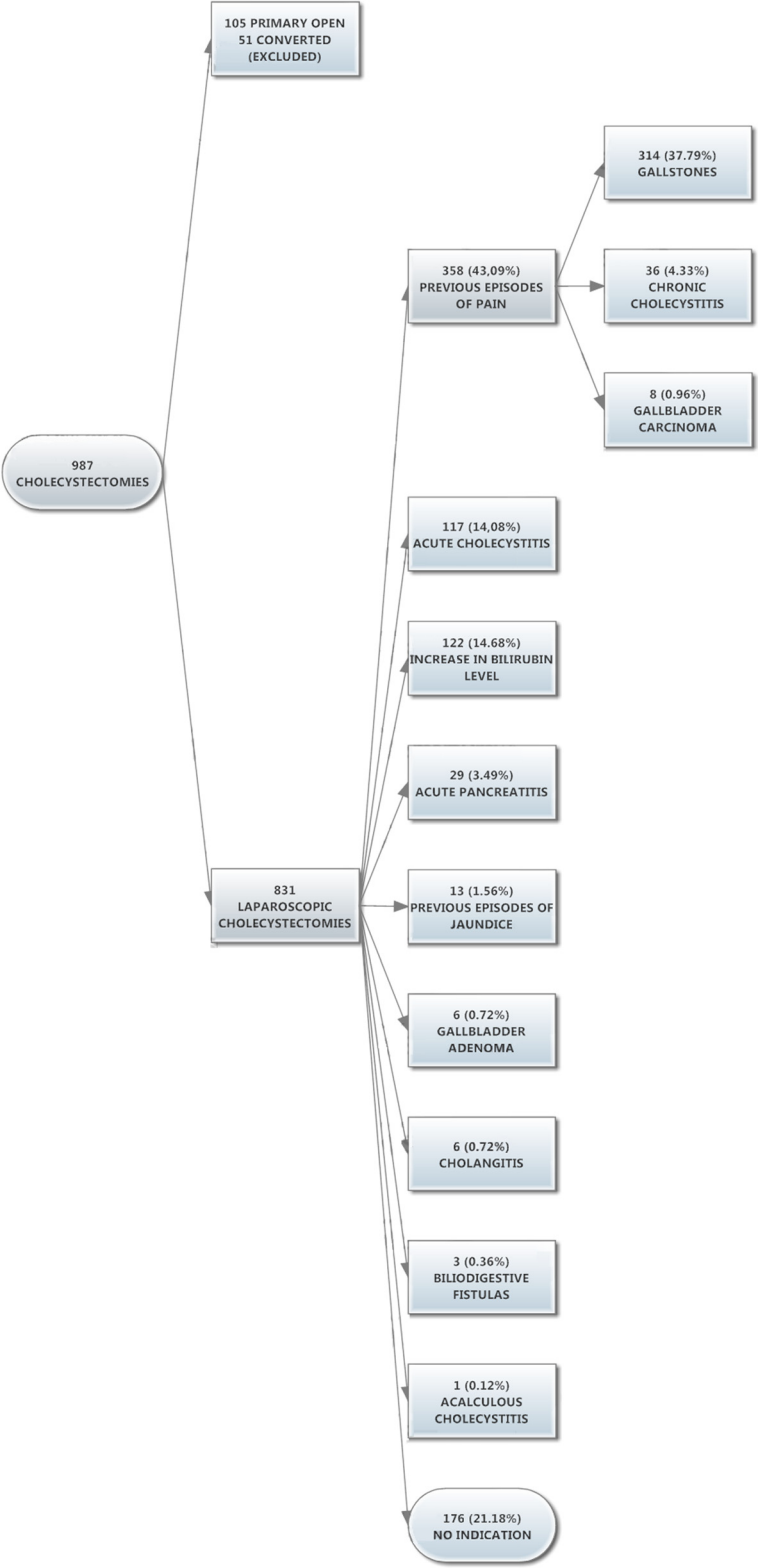
Criteria of inclusion and indications to surgery with related diagnosis at discharge.

At discharge, all patients were diagnosed as follows (Figure 2): 358 patients (43.08%) were operated on because of at least one previous episode of biliary colic before the one at admission and among these, 314 (37.79%) had simple gallstones at pathological exam, 36 (4.33%) had chronic lithiasic cholecystitis and 8 (0.96%) had gallbladder carcinoma. One hundred seventeen patients (14.08%) presented with acute lithiasic cholecystitis (defined by the presence of right upper quadrant pain with fever, raised WBC count, ultrasonographic evidence of gallstones with associated signs of inflammation). One hundred twenty-two patients (14.68%) were operated on because of an increase in bilirubin level (51 of these with radiological detection of choledocholithiasis and 26 with associated increase of WBC count). Thirteen patients (1.56%) were operated on because of a previous episode of jaundice with normal bilirubin at admission. Six patients (0.72%) had gallbladder adenomas. Six patients (0.72%) had cholangitis. Three patients (0.36%) had biliodigestive fistula and one patient (0.12%) had acalculous cholecystitis. No Mirizzi or Bouveret syndrome was detected.

By excluding all these patients, 176 patients (21.18% including 78 patients admitted only for gallstones presence diagnosed incidentally at FAST) were operated on without indication for vague symptoms not related to gallstones or for request of the patients.

The main characteristics of this subpopulation of 176 patients are reported in Table 3. Most of these patients (64.77%) were female, with a median age (25-75^th^ percentile) of 54 (44-65) years.

**Table 3:** Characteristics of patients operated on without indications

Number of patients	176
Females (%)	114 (64.77%)
Males (%)	62 (35.23%)
Median age in years (25-75th perc)	54 (44-65)
Median time to surgery in days (25-75th perc)	2 (1-5)
Median postoperative stay in days (25-75th perc)	2 (2-3)
Median overall stay in days (25-75th perc)	5 (4-9)
Diabetics	6 (3.41%)
Cirrhotics	4 (2.27%)

At follow-up, 150 of 176 patients (85,23%) were contacted by telephone (19 were unavailable and 7 died for causes unrelated to gallbladder disease) and an oral questionnaire was administered with the following results: 6 patients (4%) experienced early recurrence of pain within 30 days after the cholecystectomy; 34 patients (22,66%) experienced late recurrence of pain (30 days after operation); 107 patients (71,33%) referred no improvement in symptoms after operation, with persistence of the same preoperative vague pain whose source required further exams (e.g. gastroscopy, computed tomography, magnetic resonance imaging) to be identified. Only 3 patients (2%) referred complete disappearance of pain after operation. In conclusion, for 147 patients (98%) included in follow-up, cholecystectomy determined no early or late improvement in symptoms, confirming that these symptoms were not related to gallstones and the operation should have been avoided.

## Discussion

Immediately following the introduction of laparoscopic cholecystectomy, many authors have detected a dramatic increase in the amount of cholecystectomies performed in general hospitals. From 1990 to 1993, for instance, this increase has been approximately 30% in New York [4]. Others report the same remarkable trend during the years 1990-2001, with a peak between 1994-1998 and a subsequent stabilization through 2001 [5]. Many reasons have been proposed to explain this growth trend. Initially, it was speculated that the increase was due to a high number of patients who put off having the operation for fear of the consequences of the laparotomic approach, but then decided to be operated on given the favorable outcome related to laparoscopy [4]. However, if true, the increase of laparoscopic cholecystectomies should have been temporary and would have been extinguished in a few years concurrent with the decrease in the procrastinating patients [4]. Conversely, the increase in laparoscopic cholecystectomies has been maintained. In a very extensive review of cholecystectomies performed over time in the state of Maryland, involving more than 54 institutions, between 1985 and 1992, the annual cholecystectomy rate had risen from 1.69 per 1000 to 2.17 per 1000, representing a 28-percent increase. This increase was considered as related to the introduction of laparoscopic technique, in which younger patients, less likely to have acute cholecystitis and/or have common bile duct stones, had acquiesced to have surgery. The gained benefit was a reduction in the overall mortality of this operation from 0.84 percent in 1989 to 0.56 percent in 1992 (a 33% reduction), at the expense of an increased rate, for more benign and lesser symptomatic cases, including possible doubtful indications [12].

General indications and contraindication for performing laparoscopic cholecystectomy are well established [6]. Society of American Gastrointestinal and Endoscopic Surgeons (SAGES) guidelines for the clinical application of laparoscopic biliary tract surgery [7] indicate that conditions requiring cholecystectomy include but are not limited to cholelithiasis (when symptomatic), choledocholithiasis, biliary dyskinesia, porcelain gallbladder, acute cholecystitis and pancreatitis related to common bile duct stones. Asymptomatic gallstones are not included among the indications for laparoscopic cholecystectomy [7], although, according to Murshid et al, the distance to facility, long waiting lists in small centers, and even the unavailability of an experienced surgeon may represent an indication to perform the laparoscopic cholecystectomy in absence of symptoms [13]. Other indications for surgery in patients with asymptomatic gallstones such as advanced age, diabetes, gallbladder adenomyomatosis and risk of gallbladder cancer are still the subject of debate [14-17]. Regardless of these specific or rare conditions, we can conclude that there is general agreement that patients with asymptomatic gallstones do not require operation.

To the best of our knowledge, this is the first work which retrospectively analyzes all patients who underwent a laparoscopic cholecystectomy in order to find the percentage of patients submitted to this procedure with no indication or doubtful indications. It is remarkable that 78 patients (that is 9.38% of the cohort) were admitted and subsequently operated on only due to presence of gallstones diagnosed incidentally at FAST, without history associated symptoms. By collecting data from the diagnoses at discharge, the proportion of patients operated on with no indication resulted in an even greater percentage (176 patients who represent 21.18% of the cohort). Almost 98% of this group of patients with indefinite symptoms presented vague symptoms again after the cholecystectomy, demonstrating that the symptoms that led to cholecystectomy were not related to the presence of the gallbladder stone and determining the need of further exams to identify their source.

Speculating about the possible reasons for these data, it is important to note that gallstones are frequently considered the cause of a multitude of abdominal symptoms and, after detection, the patient is usually operated on without further investigations that, conversely, are performed only when symptoms relapse after surgery. Second, it is possible that the increased patient demand due to benefits of laparoscopy and fear of stones complications plays a determining role. Finally, the need for practice by inexperienced surgeons should be considered.

It has been proven that the risk of major bile duct injuries is still greater with laparoscopy rather than with the open approach [9,18]. By collecting data among patients operated on with doubtful of no indications, we did not detected any complications directly related to the procedure performed at our institution, but it is possible to suppose that an irresponsible increase in useless laparoscopic procedures could also result in an increase in bile duct injuries or other complications.

Finally, one question should be addressed: given the importance of surgical skills set in such a different laparoscopic procedure, how critical is the surgeon’s training in determining an increase in useless cholecystectomies? Could the constant introduction of different devices and approaches be responsible for an increase in operations performed on “easy” patients who would not otherwise need surgery?

## Conclusion

The increase in doubtful indications is recognized, and it can be considered a consequence of the new technologies introduced, an increasing in minimal access experience and demand of patients for laparoscopic surgery, with no associated modifications of the standard indications. If not prevented, this trend could continue indefinitely.

## Competing interests

All the authors declare that they have no competing interests.

## Authors’ contributions

Contributions to conception and design: IDiC, AT; Acquisition of data, EP, MM; Analysis and interpretation of data: AT, EP; Have been involved in drafting the manuscript: AT, EP; Revising it critically for important intellectual content: IDiC, MG; Have given final approval of the version to be published: IDiC. All authors read and approved the final manuscript.

## Pre-publication history

The pre-publication history for this paper can be accessed here:

http://www.biomedcentral.com/1471-2482/13/17/prepub
